# Eligibility Screening for an Early Upper Limb Stroke Rehabilitation Study

**DOI:** 10.3389/fneur.2019.00683

**Published:** 2019-07-02

**Authors:** Jeremia P. O. Held, Jannie van Duinen, Andreas R. Luft, Janne M. Veerbeek

**Affiliations:** ^1^Division of Vascular Neurology and Neurorehabilitation, Department of Neurology, University of Zurich and University Hospital Zurich, Zurich, Switzerland; ^2^Cereneo, Center for Neurology and Rehabilitation, Vitznau, Switzerland

**Keywords:** stroke, rehabilitation, trial design, screening, prospective study

## Abstract

**Introduction:** Stroke rehabilitation should start early in order to optimize patients' outcomes, but most trials include subacute or chronic patients. Although suggested that early stroke rehabilitation trials face difficulties regarding patient recruitment with corresponding low recruitment rates, no systematically collected information regarding screening and associated costs has been published. Such knowledge is essential for optimizing enrollment. Therefore, this study evaluated screening procedures for an early upper limb rehabilitation study including first-ever ischemic stroke patients <48 h after onset.

**Methods:** Screening data for a monocentric longitudinal observational cohort study was prospectively collected. Researchers screened health-care records, during the morning round and face-to-face at the stroke-unit on working days. Outcomes were the recruitment rate, reasons for non-enrollment, and screening costs.

**Results:** Over 15 months, 27 out of 845 screened ischemic stroke patients were enrolled, equaling a recruitment rate of 1.8/month. Main reasons for non-enrollment were no upper limb paresis (*N* = 456), >48 h post-stroke (*N* = 257), general comorbidity (*N* = 150), unable to follow commands (*N* = 148), and recurrent stroke (*N* = 146). Four patients were missed due to time constraints of the personnel or patient. The recruitment rate would have been 1.2 higher if also patients with recurrent strokes but without residual motor deficits or pre-stroke mRS ≥2 were considered eligible. Screening costed € 7.48 per patient.

**Discussion:** Screening at working days is sufficient to enroll patients in early stroke rehabilitation trials. Inclusion criteria regarding recurrent strokes should be less stringent to boost recruitment rates without increasing bias. Multicenter collaborations are needed to finish well-powered early stroke rehabilitation studies within a reasonable time.

**Ethics and Study Registration:** Authorization from the local ethical committee was not required, as this study does not fall within the scope of the Human Research Act (BASEC Identifier: Req-2017-00844). The project was registered at http://www.clinicaltrials.gov (Identifier: NCT03633422).

## Introduction

It is assumed that rehabilitation should be initiated early after stroke in order to optimize stroke outcomes (1). However, stroke rehabilitation studies have traditionally been performed in patients beyond the first 4–12 weeks post-stroke (2). Therefore, it has been stressed that future stroke rehabilitation trials should include patients early after symptom onset (2).

The change toward “acute” stroke rehabilitation trials has major consequences for clinical trial design and particularly for participant screening procedures. However, little information is available regarding screening procedures for these early initiated trials, including recruitment rates, exclusion reasons, and costs. It is likely that recruitment rates will be low, as diagnostic procedures and medical interventions are first priority early after stroke. A recent review by Feldman et al. (3) supports the expected difficulties for patient recruitment in very early stroke rehabilitation trials. They showed that the development of recruitment rates for acute medical stroke trials has been sobering during the last 20 years: from 1990 to 2004, 0.41 participants were enrolled per site per month and between 2010 and 2014, this number has decreased to 0.26. Researchers of the largest randomized controlled trial in stroke rehabilitation, the A Very Early Rehabilitation Trial (AVERT), had to screen 25,237 patients at 56 stroke units in five countries over an 8 year time course to enroll 2'104 patients (4) and this underlines the difficulties to recruit patients early after stroke. The trial flow provides some information regarding the exclusion reasons, but not in detail and for about one quarter of the non-eligible patients, the reason remained unclear. The monocentric observational SALGOT-study had better recruitment rates—over 18 months, 117 out of 763 screened first-ever stroke patients were enrolled <72 h post-stroke (5) and the researchers provided more details regarding the selection process. However, the applied screening procedures and their associated costs remained unclear.

Knowledge regarding screening procedures for early stroke rehabilitation trials is essential for optimizing the design and completion of stroke rehabilitation trials (6) and with that, timely answering questions regarding post-stroke recovery and the efficacy of early rehabilitation interventions. Therefore, our main aim was to describe the recruitment rate for an early longitudinal observational upper limb rehabilitation study post-stroke (i.e., <48 h), including a detailed analysis of exclusion reasons. The secondary aims were to gain insight in time investment and costs associated with eligibility screening.

## Methods

### Definition

In the present work, the following definition was applied for screened patients: “Screened patients could broadly be defined as patients with the disease who present at the site(s) during the recruitment time interval, including those who were not formally assessed for eligibility” (7).

### Study Design

The screening procedures for a monocentric, prospective observational cohort study were evaluated (ClinicalTrials.gov identifier NCT03633422). The main goal of the cohort study was to externally validate the Shoulder Abduction Finger Extension (SAFE) model (8) within 48 h after stroke for predicting outcome of upper limb capacity 3 months after symptom onset. The study's inclusion criteria were (1) a first-ever unilateral ischemic stroke <48 h confirmed by diffusion-weighted magnetic resonance imaging and/or computed tomography; (2) age ≥18 years; (3) able to follow one-staged commands; (4) National Institutes of Health Stroke Scale (NIHSS) arm score of ≥1; and (5) informed consent. Main exclusion criteria were (1) pre-stroke modified Rankin Scale score of >2; (2) neurological or other diseases affecting the upper limb(s) before stroke; (3) intravenous line in the upper limb(s) limiting assessment; (4) contra-indications on ethical grounds (such as palliative stroke care, imprisonment); and (5) expected or known non-compliance (such as alcohol and drug abuse).

The estimated number of patients to be enrolled in 1 year was 40, equaling a monthly recruitment rate of 3.3. Recruitment started on the 01/10/2017 at the University Hospital Zurich, Department of Neurology, Zurich, Switzerland. The acute stroke unit has eight beds and a yearly admission rate of about 800 acute stroke patients in the period 2010–2016. For the present work, data obtained until 31/12/2018 were considered.

For evaluating screening procedures, authorization from the local ethical committee was not required, as this does not fall within the scope of the Swiss Human Research Act (BASEC identifier Req-2017-00844). The prospective cohort study on which we applied the evaluation of screening procedures was approved by the cantonal ethics committee Zurich (BASEC identifier Req-2017-00889; ClinicalTrials.gov identifier NCT03287739).

### Screening Procedures and Outcomes

Screening was performed by one of the researchers from Monday to Friday during the morning round, twice a day in electronic files, and if indicated, face-to-face on the ward. Screening on the ward was performed when the stroke patient was, based on descriptive reports, likely to be eligible. The following data was prospectively collected: number of patients screened; reasons for non-enrollment; number of enrolled patients; gender; NIHSS at screening; the application of thrombolysis and thrombectomy; time needed for each patient identification method (5 min blocks); and number of patients screened on the ward. Main outcomes were the recruitment rate, defined as the number of patients enrolled per month of recruitment (9), and frequency of each of the exclusion reasons. Secondary outcomes were the time investment associated with screening expressed as minutes per day and corresponding personnel costs in Euros.

### Analysis

Recruitment rate and reasons for exclusion were analyzed by descriptive statistics. Time needed for screening was calculated by summing the minutes spent on each identification method divided by the number of screening days. Costs associated with screening one patient was calculated as follows:

$$Costs=\;\frac{\left(\text{Total screening time }\left[\text{hours}\right]*\;€\;49.37\right)}{\text{Number of screened patients}}$$

We furthermore calculated the screening costs associated with enrolling one patient:

$$Costs=\;\frac{\left(\text{Total screening time }\left[\text{hours}\right]*\;€\;49.37\right)}{\text{Number of enrolled patients}}$$

Analyses were performed with IBM SPSS Software version 25 (IBM Analytics).

## Results

Eight hundred forty-five patients were screened in 15 months, out of which 110 were screened on the ward (see Table 1). During screening on the ward, the following criteria had to be checked for: NIHSS arm score, the ability to follow one-staged commands and the presence of intravenous lines hampering assessment. Patient characteristics can be found in Table 2. Two patients fulfilled all eligibility criteria, except for consent to participate. Thirty-one patients were eligible out of which three could not be enrolled due to time constraints of the study personnel and one due to a full patient schedule. Thus, the actual recruitment rate amounted 1.8 per month, but would have been 2.1 if the four patients fulfilling the criteria but were not enrolled had participated.

**Table 1:** Screening characteristics.

Patients screened	
Ischemic stroke patients screened in total, *N*	845
Ischemic stroke patients screened on ward, *N*	110
Eligible ischemic stroke patients, *N* (%)	31 (3.8%)
Included, *N* (%)	27 (3.2%)
Not included due to time constraints researchers, *N*	3
Not included due to time constraints patient, *N*	1
Patients screened >48 h, *N*	257
Due to absence researchers, *N* (%)	181 (70.4%)
Fulfilled all other criteria, *N* (%)	0 (0.0%)
Due to late admission, *N* (%)	76 (29.6%)


**Table 2:** Patient characteristics.

Gender, female/male^*^	366 (43.3%)/479 (56.7%)
Thrombolysis, yes/no^*^	223 (26.4%)/611 (72.3%)
Thrombectomy, yes/no^*^	215 (25.4%)/619 (73.3%)
Length of hospital stay, days^†^	8 (4–12)
NIHSS	
Total admission^†^	5 (2–12)
Arm admission^†^	1 (0–3)
Arm admission ≥1, yes/no^*^	459 (54.3%)/385 (45.6%)
Arm screening ≥1, yes/no^*^	388 (45.9%)/456 (54.0%)
Recovered NIHSS arm from admission to screening^^*^^^‡^	97 (21.1%)
Thrombolysis, yes/no^*^	44 (45.4%), 50 (51.5%)
Thrombectomy, yes/no^*^	23 (23.7%), 71 (73.2%)
Not recovered NIHSS arm from admission to screening^^*^§^	362 (78.9%)
Thrombolysis, yes/no^*^	108 (29.8%)/250 (69.1%)
Thrombectomy, yes/no^*^	143 (39.5%)/215 (59.4%)

NIHSS, National Institutes of Health Stroke Scale;

* , N (%);

† , median (interquartile range);

‡ , NIHSS arm ≥1 at admission to 0 at screening;

§ *, NIHSS arm ≥1 at admission and at screening. Percentages not counting up to 100 reflect the percentage of missing data*.

**Figure 1:**
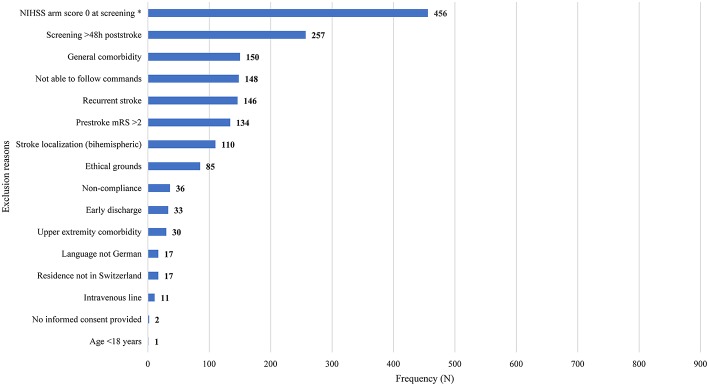
Overview of exclusion reasons for non-eligible patients (*N* = 814). Exclusion reasons arranged in descending order based on their prevalence. Stroke localization (bihemispheric) and recurrent stroke refer to the inclusion criterion “first-ever unilateral ischemic stroke.” The criterion “comorbidity” was divided into general comorbidity (e.g., Morbus Parkinson) and specific upper limb comorbidity (e.g., arthrosis of the hand, humerus fracture). Patients not residing in Switzerland could not be enrolled, as they would not be able to return for the follow-up visits. Four patients fulfilling the criteria could not be enrolled due to time constraints of researchers (*N* = 3) or patient (*N* = 1). h, hours; NIHSS, National Institutes of Health Stroke Scale; mRS, modified Rankin Scale; *One data point missing.

**Figure 2:**
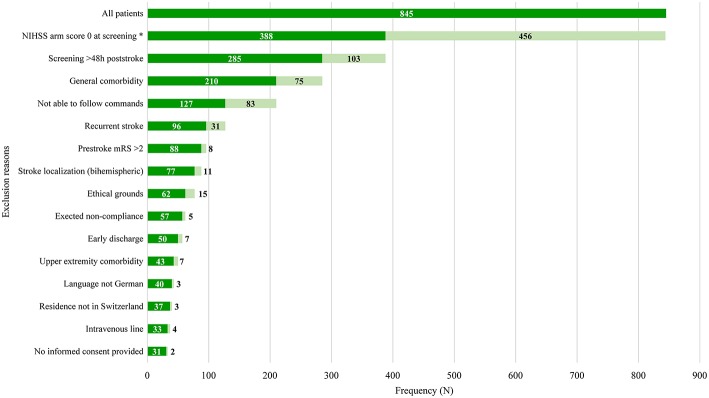
Overview of cumulative exclusion reasons. Light-green bars indicate the number of patients fulfilling all previous criteria but not the current criterion. Dark-green bars indicate number of patients fulfilling all previous criteria including the current criterion. Exclusion reasons arranged in ascending order based on their prevalence. Stroke localization (bihemispheric) and recurrent stroke refer to the inclusion criterion “first-ever unilateral ischemic stroke.” The criterion “comorbidity” was divided into general comorbidity (e.g., Morbus Parkinson) and specific upper limb comorbidity (e.g., arthrosis of the hand, humerus fracture). Patients not residing in Switzerland could not be enrolled, as they would not be able to return for the follow-up visits. h, hours; NIHSS, National Institutes of Health Stroke Scale; mRS, modified Rankin Scale; *One data point missing.

Non-eligible patients had a median of two (interquartile range 1–3) exclusion reasons. The main exclusion reasons were an NIHSS arm score of zero at screening (*N* = 456, 54%), >48 h post-stroke (*N* = 257; 30.4%), general comorbidity (*N* = 150; 17.8%), unable to follow one-staged commands (*N* = 148; 17.5%), and a recurrent stroke (*N* = 146; 17.3%). Please see Figure 1 for an overview of all exclusion reasons and Figure 2 for a cumulative presentation of the exclusion reasons. To provide more insight in the exclusion reasons, we subdivided “(1) a first-ever unilateral ischemic stroke <48 h confirmed by diffusion-weighted magnetic resonance imaging and/or computed tomography” into stroke localization (bihemispheric) and recurrent strokes, and “comorbidity” into general comorbidity (e.g., Morbus Parkinson), and specific upper limb comorbidity (e.g., arthrosis of the hand or humerus fracture). Patients not residing in Switzerland could not be included, as they would not be available for the follow-up visit at 3 months post-stroke, the main outcome time point of the longitudinal cohort study.

Ninety-seven (21.3%) of the patients with an NIHSS arm score of zero at screening had an NIHSS arm score of one or more points at hospital admission. Reasons for screening patients >48 h post-stroke was due to absence of study personnel in 70.4% and late admission in 29.6% of the cases. Absence of study personnel referred to weekends, (public) holidays and conference attendance. None of these late-screened patients fulfilled the other study criteria.

*Post-hoc* analysis showed that 18 patients with a recurrent stroke did not have any other exclusion reason and 13 of them had no residual motor deficits. Removing the mRS as an inclusion criterion for our upper limb rehabilitation study would have led to five more patients being eligible.

Daily screening time amounted 9.15 min for electronic records, 14.24 min for morning rounds, and 4.55 min on the ward (Table 3). Screening one patient took on average 9.09 min, which costed € 7.48. To enroll one patient, 264.83 min had to be screened, costing € 217.91.

**Table 3:** Time investment and costs related to screening.

Total number of screening days, *N*	275
Total screening time, minutes/day	27.93
Electronic health-care records, minutes/day	9.15
Morning rounds, minutes/day	14.24
Ward, minutes/day	4.55
Minutes screened/patient	9.09
Minutes screened/enrolled patient	264.83
Costs per screened patient, €	7.48
Costs per enrolled patient, €	217.91

## Discussion

Prospectively observing eligibility screening procedures for an early upper limb motor rehabilitation study showed that about two stroke patients a month could be enrolled in our center, although the target was 3.3. An NIHSS arm score of zero was by far the most frequently observed exclusion reason, followed by screening >48 h post-stroke, the presence of interfering general comorbidities, unable to follow commands, and a recurrent stroke. The applied screening procedures took about 9 min per patient and costed € 7.48. The screening costs per enrolled patient amounted € 217.91, excluding the consent process.

With 1.8 per month, the enrolment rate in our center is higher than the recruitment rates per center of early multicenter rehabilitation trials like the randomized controlled AVERT-study (0.38) (4) and observational EPOS-study (0.74) (8), but lower than the observational monocentric SALGOT-study (6.5) (5). A relatively high enrollment rate in monocentric studies could be attributed by the full-time presence of dedicated, experienced research personnel as a driving force behind patient inclusion and alternative consent procedures (10). However, it is not surprising that the total number of enrolled patients per month for monocentric studies was lower than for multicentric studies. Considering our recruitment rate and that a high number of patients are needed for well-powered upper limb randomized trials (11), multiple centers should be involved to complete the study within an acceptable timeframe. Furthermore, our recruitment rate could have been 0.9 per month higher if also patients with recurrent strokes without residual motor deficits were deemed eligible (i.e., 13 more patients would have been eligible). Another possibility to increase the recruitment rate for upper limb trials is by deciding against taking a pre-stroke mRS score as an inclusion criterion, as long as global disability is not the main outcome. A higher mRS score does not necessarily reflect limitations in upper limb function prior to stroke. In our case, this would have led to five more patients being eligible, i.e., the recruitment rate would have been 0.3 higher. In addition, including hemorrhagic stroke patients, like done in the SALGOT-study (5), could have improved the recruitment rate. Strategies to increase enrollment rates for studies with chronic stroke patients, such as referral through health-care provides, advertisement, or postal invitations (12–15) are unfortunately not transferable to acute studies. We expected that early recruitment would be hampered by early medical diagnostics and interventions, but only one patient could not be included due to a full schedule, mainly related to a diagnostic work-up.

The lack of an upper limb paresis was the main reason for non-enrollment; 54.3% of the patients had an NIHSS arm score of ≥1 at hospital admission and 21.3% of them had a fully recovered arm at screening. The recent SALGOT-study reported a similar prevalence within 72 h post-stroke (16). This is interesting, as a few decades ago, prevalence estimates of upper limb paresis up till 80% were reported (17). Acknowledging that most prevalence data are obtained before the implementation of thrombolysis and endovascular treatment (18), it could be hypothesized that the availability of effective early medical interventions might have led to the fast recovery of arm paresis early after symptom onset and as a consequence a reduced number of eligible patients. The difference could also be a result from how we objectified upper paresis. The NIHSS assesses strength of the proximal upper limb on a 5-point scale, ranging from “0” (normal strength) to “4” (no movement). However, a maximal score of zero does not mean that there could not be a strength deficit in the more distal part of the arm. In addition, muscle contraction against resistance is not tested. Therefore, the Motricity Index (19) or the SAFE-score (8) are better screening tools, as they allow quickly measuring both the proximal and distal upper limb and do consider resistance. The Action Research Arm Test or Fugl-Meyer Assessment are even more likely to show motor limitations (16), but also require much more time.

With 30.4% of the patients screened beyond 48 h after onset, the present study also showed that including patients early post-stroke is challenging. Twenty-nine point six percent of these patients were admitted >48 h, the other 70.4% was screened too late due to absence of study personnel. Fortunately, none of the latter fulfilled the other inclusion criteria, although this could be the case, as patients admitted between Friday afternoon and Saturday morning are outside the 48 h timeframe on Monday morning. This implies that eligibility screening for early rehabilitation trials does not necessarily require availability of research personnel in the weekend, unless the period for inclusion is restricted to the first 24 to 36 h post-stroke.

The main limitation of this work is that it refers to a monocentric study, which might hamper generalizing its findings.

## Summary/Conclusions

Our work highlighted the efforts needed to screen patients for early stroke rehabilitation trials and the associated costs. Screening on working days by dedicated research personnel should be sufficient as long as patients can be enrolled up to at least 48 h after symptom onset. However, multiple centers are required to finish well-powered early stroke rehabilitation trials with an acceptable timeframe. For upper limb trials, screening by the NIHSS arm item might be too global and results in non-enrollment of patients who have a more distal paresis. Furthermore, the inclusion of patients with a recurrent stroke should be considered, as long as they have no residual deficits, or those with a pre-stroke mRS of >2 but without comorbidities affecting the upper limb, as well as hemorrhagic strokes. Therewith, both recruitment rates and generalizability could be improved, and study duration and corresponding costs decreased. Finally, we advocate that future trialists describe their reasons for non-enrollment into more detail, as this helps the readers critically judge the transfer of the results as well as other early stroke rehabilitation trialists in designing their inclusion and exclusion criteria more carefully.

## Data Availability

The raw data supporting the conclusions of this manuscript will be made available by the authors, without undue reservation, to any qualified researcher.

## Ethics Statement

Authorization from the local ethical committee was not required, as this study does not fall within the scope of the Human Research Act (BASEC Identifier: Req-2017-00844).

## Author Contributions

JH and JV participated in the experimental design. JH, JvD, and JV were responsible for data acquisition. JV was responsible for drafting and adapting the manuscript and analyzed the data. Data interpretation was done by JH and JV. JH, JvD, and AL were responsible for critically revising the manuscript. All authors approved the final draft of the manuscript and agreed to be accountable for all aspects of the work in ensuring that questions related to the accuracy or integrity of any part of the work are appropriately investigated and resolved.

### Conflict of Interest Statement

The authors declare that the research was conducted in the absence of any commercial or financial relationships that could be construed as a potential conflict of interest.

## References

[1] Murphy TH Corbett D. Plasticity during stroke recovery: from synapse to behaviour. Nat Rev Neurosci. (2009) 10:861–72. 10.1038/nrn2735 19888284

[2] Stinear C Ackerley S Byblow W Rehabilitation is initiated early after stroke, but most motor rehabilitation trials are not: a systematic review. Stroke. (2013) 44:2039–45. 10.1161/strokeaha.113.000968 23715959

[3] Feldman WB Kim AS Chiong W Trends in recruitment rates for acute stroke trials, 1990–2014. Stroke. (2017) 48:799–801. 10.1161/strokeaha.116.014458 28104835 PMC5330837

[4] AVERT Trial Collaboration Group Efficacy and safety of very early mobilisation within 24 h of stroke onset (AVERT): a randomised controlled trial. Lancet. (2015) 386:46–55. 10.1016/S0140-6736(15)60690-0 25892679

[5] Persson HC Opheim A Lundgren-Nilsson A Alt Murphy M Danielsson A Sunnerhagen KS. Upper extremity recovery after ischaemic and haemorrhagic stroke: part of the SALGOT study. Eur Stroke J. (2016) 1:310–9. 10.1177/2396987316672809 31008293 PMC6301248

[6] Kasenda B von Elm E You J Blumle A Tomonaga Y Saccilotto R. Prevalence, characteristics, and publication of discontinued randomized trials. JAMA. (2014) 311:1045–51. 10.1001/jama.2014.1361 24618966

[7] Elm JJ Palesch Y Easton JD Lindblad A Barsan W Silbergleit R. Screen failure data in clinical trials: are screening logs worth it? Clin Trials. (2014) 11:467–72. 10.1177/1740774514538706 24925082 PMC4264995

[8] Nijland RH Van Wegen EE Harmeling-Van der Wel BC Kwakkel G. Presence of finger extension and shoulder abduction within 72 hours after stroke predicts functional recovery: early prediction of functional outcome after stroke: the EPOS cohort study. Stroke. (2010) 41:745–50. 10.1161/STROKEAHA.109.572065 20167916

[9] Elkins JS Khatabi T Fung L Rootenberg J Johnston SC. Recruiting subjects for acute stroke trials: a meta-analysis. Stroke. (2006) 37:123–8. 10.1161/01.STR.0000195149.44390.aa 16322489

[10] Berge E Stapf C Al-Shahi Salman R Ford GA Sandercock P Van der Worp HB. Methods to improve patient recruitment and retention in stroke trials. Int J Stroke. (2016) 11:663–76. 10.1177/1747493016641963 27118766

[11] Winters C Heymans MW Van Wegen EE Kwakkel G. How to design clinical rehabilitation trials for the upper paretic limb early post stroke? Trials. (2016) 17:468. 10.1186/s13063-016-1592-x 27669893 PMC5037599

[12] Taylor-Piliae RE Boros D Coull BM. Strategies to improve recruitment and retention of older stroke survivors to a randomized clinical exercise trial. J Stroke Cerebrovasc Dis. (2014) 23:462–8. 10.1016/j.jstrokecerebrovasdis.2013.03.031 23643477

[13] Lloyd G Dean CM Ada L. Issues in recruiting community-dwelling stroke survivors to clinical trials: the AMBULATE trial. Contemp Clin Trials. (2010) 31:289–92. 10.1016/j.cct.2010.04.003 20435166

[14] Mares K Cross J Clark A Vaughan S Barton GR Poland F. Feasibility of a randomized controlled trial of functional strength training for people between six months and five years after stroke: festivals trial. Trials. (2014) 15:322. 10.1186/1745-6215-15-322 25118156 PMC4138387

[15] Treweek S Pitkethly M Cook J Fraser C Mitchell E Sullivan F. Strategies to improve recruitment to randomised trials. Cochrane Database Syst Rev. (2018) 2:Mr000013. 10.1002/14651858.MR000013.pub6 29468635 PMC7078793

[16] Persson HC Parziali M Danielsson A Sunnerhagen KS. Outcome and upper extremity function within 72 hours after first occasion of stroke in an unselected population at a stroke unit. A part of the SALGOT study. BMC Neurol. (2012) 12:162. 10.1186/1471-2377-12-162 23273107 PMC3554428

[17] Rathore SS Hinn AR Cooper LS Tyroler HA Rosamond WD. Characterization of incident stroke signs and symptoms: findings from the atherosclerosis risk in communities study. Stroke. (2002) 33:2718–21. 10.1161/01.STR.0000035286.87503.31 12411667

[18] Berkhemer OA Fransen PS Beumer D Van den Berg LA Lingsma HF Yoo AJ. A randomized trial of intraarterial treatment for acute ischemic stroke. N Engl J Med. (2015) 372:11–20. 10.1056/NEJMoa1411587 25517348

[19] Collin C Wade D. Assessing motor impairment after stroke: a pilot reliability study. J Neurol Neurosurg Psychiatry. (1990) 53:576–9. 2391521 10.1136/jnnp.53.7.576 PMC488133

